# A systematic review on the use of action research methods in mental health nursing care

**DOI:** 10.1111/jan.15463

**Published:** 2022-10-27

**Authors:** Antonio R. Moreno‐Poyato, Martí Subias‐Miquel, Diana Tolosa‐Merlos, Ana Ventosa‐Ruiz, Alonso Pérez‐Toribio, Kadhija EL Abidi, Raquel Navarro‐Maldonado, Raquel Suárez‐Pérez, Rosario Valera‐Fernández, Maria Romeu‐Labayen, Teresa Lluch‐Canut, Juan Roldán‐Merino, Montserrat Puig‐Llobet

**Affiliations:** ^1^ Department of Public Health, Mental Health and Maternal and Child Health Nursing, Nursing School Universitat de Barcelona L'Hospitalet de Llobregat Spain; ^2^ Community Mental Health Center (CMHC) Ciutat Vella, Parc Sanitari Sant Joan de Déu Sant Boi de Llobregat Spain; ^3^ Nursing and Health Doctoral Programme, Nursing School Universitat de Barcelona Barcelona Spain; ^4^ Institut de Neuropsiquiatria i Addiccions Hospital del Mar Barcelona Spain; ^5^ Hospital Benito Menni Complejo Asistencial Sant Boi de Llobregat Spain; ^6^ Unitat de Salut Mental de l'Hospitalet, Servei d'Atenció Primària Delta de Llobregat, Direcció d'Atenció Primària Costa de Ponent Institut Català de la Salut L'Hospitalet de Llobregat Spain; ^7^ Gynecology and Obstetrics Unit Hospital Can Misses Ibiza Spain; ^8^ Nursing and Health Doctoral Programme, Nursing Department, Faculty of Nursing Universitat Rovira i Virgili Tarragona Spain; ^9^ Centro de Salud de Zarracina, Servicio de atención primaria, Servicio de Salud del Principado de Asturias (SESPA) Oviedo Spain; ^10^ AFIN Research Group and Outreach Centre Autonomous University of Barcelona Cerdanyola del Vallès Spain; ^11^ Campus Docent Sant Joan de Déu Fundació Privada, School of Nursing University of Barcelona Barcelona Spain

**Keywords:** action research, mental health, nursing care, systematic review

## Abstract

**Aims:**

To identify and synthesize evidence on the use of action research methods in mental health nursing care.

**Design:**

Systematic review.

**Data Sources:**

CINAHL, Web of Science, PubMed and Scopus databases were searched in January 2021.

**Review Methods:**

Data were selected using the updated Preferred Reporting Items for Systematic Reviews and Meta‐Analysis framework. Two reviewers independently conducted the study selection, and quality appraisal using Joanna Briggs Institute Critical Appraisal Checklist for Qualitative Research, data extraction and data analysis procedures.

**Results:**

Sixteen studies, half of which used participatory action research, were included in this review. Nurses, along with other stakeholders, were an active part of the action research process. The main topics of interest addressed were categorized as improving the adoption of a person‐centred approach to care and improving decision‐making procedures. The use of action research helped the participants to identify the meaning they attached to the topic of interest to be improved. Moreover, this method helped to identify needs and strategies for improving care. The studies concurred that the use of action research enabled participants to gain awareness, improve attitudes and acquire knowledge. In addition, it enabled participants to gain confidence and security in the group context, as key aspects of their empowerment.

**Conclusion:**

This review shows the usefulness of action research in any mental health nursing context, contributing to the improvement of care at both the individual and collective levels.

**Impact:**

This paper demonstrates the use of the action research method in the field of mental health nursing. Its use has improved the clinical practice of nurses as well as that of teams in both community and hospital settings, addressing issues of the person‐centred approach to care and decision‐making procedures.

## 1 INTRODUCTION

Action research is a research method that facilitates understanding and improving the world by transforming it (Kemmis & Mctaggart, 2008). Traditionally, this type of approach has been used in the social sciences (Rowell et al., 2017), although in recent years it is also been successfully used internationally in the area of health sciences (Cordeiro & Soares, 2018) and, specifically, in the field of nursing (Effendy et al., 2022). In fact, action research has been shown to be useful for implementing evidence‐based practice (Munten et al., 2010) in the area of nursing education or for improving nursing practices in intensive care (Soh et al., 2011). However, no evidence has been found to demonstrate its use and utility, specifically in the field of mental health nursing.

### 1.1 Background

The action research method relies on inquiry to understand and improve the practices in which one participates and the situations in which one finds oneself (Baum et al., 2006; Kemmis & Mctaggart, 2008). This method includes a family of related approaches that integrate theory and action, approaches that are adapted to the broad and complex contexts and problems addressed (e.g., participatory action research; collaborative action research; cooperative inquiry; feminist participatory action research; rural participatory research and critical participatory action research) (Bradbury, 2015). The action research process consists of a continuous and cyclical interaction where each cycle comprises different stages, such as action, reflection and evaluation (Rowell et al., 2017). This process enables a better understanding of the nature of practices, their purpose and the necessary conditions to modify these, ultimately resulting in social transformation (Bradbury et al., 2019). The process can be individual or collective and can modify or produce changes at the level of the individual, collective or organization (Kemmis & Mctaggart, 2008; Rowell et al., 2017).

It is evident that the nature of the action research process contributes to its potential as a method for improving work practices in complex contexts such as healthcare. Thus, there are some reviews in the international literature that have sought to examine the use of action research in the healthcare context. Waterman et al. (2001) conducted a systematic review aimed at identifying research projects in the healthcare field of the UK. The authors concluded that action research had the potential to be useful, not only in areas such as innovation development, healthcare improvement and knowledge development but also to facilitate practitioner understanding and user and professional engagement (Waterman et al., 2001). More recently, Cordeiro and Soares (2018) conducted a scoping review of the international literature to explore how action research had also been applied in studies framed in the general health context. These authors concluded that action research was useful in any context where there were organizational and/or political problems, as well as for addressing gaps in health education at the collective and individual levels (Cordeiro & Soares, 2018).

Specifically, in the area of nursing, nurses are constantly concerned about the difficulties related to the gap between theory, research and practice, therefore, it is necessary to translate these theoretical concepts into practice through research (Greenway et al., 2019). In this sense, Munten et al. (2010) evaluated the use of action research in the implementation of Evidence‐Based Practice. The authors concluded that action research was a promising method in what appears to be a useful way to bridge the gap between nursing research and practice (Munten et al., 2010). More specifically, a review was published on the use of action research in the intensive care units. (Soh et al., 2011). In this study, the authors also concluded that action research was a promising method to address the improvement of clinical practice in the intensive care units, although they detected shortcomings in the reviewed studies in terms of clinical outcome assessment (Soh et al., 2011). Likewise, Cusack et al. (2018) studied the value of action research in public health nursing practice, concluding that action research should be the method of choice for examining complex and deeply rooted nursing issues in the profession. In any case, most of the reviews point to the lack of publications about the use of action research and the lack of synthesis about the methodology used (Cordeiro & Soares, 2018; Cusack et al., 2018; Soh et al., 2011; Waterman et al., 2001).

This is also the case in mental health nursing. Over the last few years, multiple published studies have used the action research method with different methodological approaches aimed at improving different aspects of clinical practice, such as patient safety (Kanerva et al., 2017), mental health nursing skills (Kelly et al., 2019), self‐harm in young people (Bailey et al., 2019) or mental health care programs (Lin et al., 2018; Rezaie & Phillips, 2020). There is great heterogeneity in the studies to date about the description of the methodological approaches and the results obtained (Bailey et al., 2019; Kanerva et al., 2017; Kelly et al., 2019; Rezaie & Phillips, 2020). To our knowledge, no studies have performed a comprehensive analysis and synthesis of the use of action research in mental health nursing. Therefore, it is necessary to compile the available evidence. These findings could provide new knowledge to aid future research aiming to use this method to transform and improve mental health nursing practice.

## 2 THE REVIEW

### 2.1 Aims

The objective of this systematic review was to locate and describe the best available evidence related to the form and content in which the action research method has been applied in the context of mental health nursing. Hence, this review aimed to answer the following questions:

- What is the type of action research methodology used in mental health nursing and who participated in the research?
- What are the main dimensions addressed as a focus for change or improvement in studies that have used the action research method?
- What new knowledge and plans for action or social change do the studies provide?

### 2.2 Design

A systematic review and thematic synthesis of qualitative studies were performed. This review is presented following The Preferred Reporting Items for Systematic Reviews and Meta‐Analyses (PRISMA) guidelines. The revision was registered with PROSPERO on March 25, 2021, with registration number CRD42021238794.

### 2.3 Search methods

The following steps were followed in this review. After a preliminary search to locate any existing or planned systematic reviews on this topic, the first step was a limited search of the PubMed and CINAHL databases using terms related to action research and mental health nursing. An analysis of the text words contained in the title, abstract and index terms used to describe the articles retrieved during the search was then performed. Based on this, an adapted search strategy was designed for all other databases in which searches were performed using database‐specific keywords and subject headings, where appropriate. The search strategies can be found in Data S1. The searches were limited to studies published in English from 2009 to 2020. The following databases were searched: CINAHL, Web of Science, PubMed and Scopus. The inclusion criteria were:

- Types of participants: Nurses in clinical or management roles and/or patients or health care recipients who had participated in action research.
- Types of intervention(s)/phenomena of interest: Studies that used action research as a research method and aimed to improve and/or change care and/or management in the context of mental health nursing, both community and hospital settings.
- Types of studies: qualitative primary research that illustrated the action research methodology and methods used, the research process and the results or findings (e.g., knowledge creation, problem‐solving, action and others). Studies that reported only one phase or incomplete reports about the action research process were not considered.

### 2.4 Search outcomes

**FIGURE 1:**
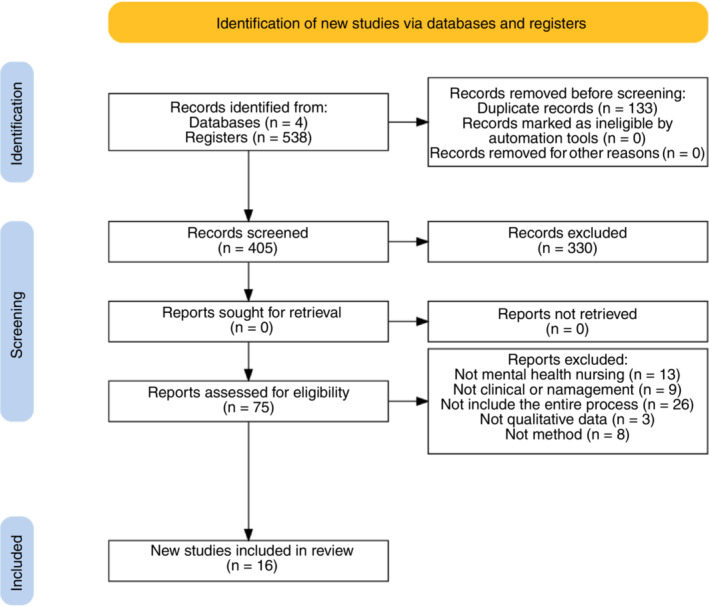
PRISMA flow diagram.

The preliminary search yielded 538 records. Subsequently, 133 duplicate records were removed, leaving 405 records to be filtered based on their titles and abstracts. In total, 75 full‐text articles were assessed for eligibility and subsequently, of which 59 records were excluded. Of those excluded at this stage, 13 did not pertain to mental health nursing, 9 were not directed to the clinical or management setting, 8 did not pertain to the action research method, 26 failed to capture the entire process and three did not present qualitative data. As a result, 16 articles met the criteria and were included in this review. Figure 1 illustrates the search method and the selection process.

### 2.5 Quality appraisal

Prior to analysis, the selected articles were assessed using the Joanna Briggs Institute Critical Appraisal Checklist (JBI‐QARI) to evaluate the quality of individual studies. Two reviewers assessed the studies independently. Disagreements between the two reviewers were resolved by consensus or by consulting a third reviewer when necessary. Although some studies did not report all the items collected in the JBI‐QARI, the team decided not to exclude any of them since the purpose of the quality assessment was to follow a systematic and standardized process that would highlight the quality of the available evidence on the study phenomenon (Vanderspank‐Wright et al., 2018). The results of the quality assessment are included in Data S2.

### 2.6 Data abstraction

Initially, a standardized data extraction form was developed that included the following fields: Author, years and source origin, Objective/s, Participants, Concept/Context, Method/Cycles and stages/Collection techniques/Duration, Knowledge building and Social change. Using this form, two authors independently extracted data. Disagreements were resolved by consensus or by consulting a third reviewer.

### 2.7 Synthesis

The data were analysed using thematic content analysis (Crowe et al., 2015). During the first stage, the text obtained was fragmented into descriptive codes that were assigned according to their semantic content. In the second stage, these codes were grouped into more analytical sub‐themes, classifying the codes according to the meaning of the linguistic units and their combinations. Finally, in a third hierarchical stage, the semantic analysis of the previous subthemes was considered, and the codes were classified deductively according to the research questions of the study.

## 3 RESULTS

### 3.1 Characteristics of included studies

The characteristics of the 16 included studies are shown in Table 1. Many of the included studies were conducted in the United Kingdom (*k* = 6), followed by Australia (*k* = 2), whereas only one study was included from the following countries: USA, Canada, Ireland, Norway, Sweden, Finland, Spain and Brazil. About the setting in which the studies were carried out, nine were conducted in inpatient care, and seven in community care. In terms of the participants, 14 of the studies involved nurses on their own or together with other professionals and patients. However, only half of the studies reviewed involved patients (Chambers et al., 2013; Chandley et al., 2014; Clements, 2012; Hutchinson & Lovell, 2013; Kidd et al., 2015; Lange, 2011; Larkin et al., 2015; Onnela et al., 2014) and there was mixed participation of users and nurses in only five studies (Chambers et al., 2013; Chandley et al., 2014; Kidd et al., 2015; Larkin et al., 2015; Onnela et al., 2014). As for the number of participants, this ranged from eight to 45 people in most studies. However, one study mentions the collaboration of over 150 people (Larkin et al., 2015), whereas two of the studies did not specify the number of participants (Hyde et al., 2009; Onnela et al., 2014).

**TABLE 1:** Action research studies in mental health nursing care

Author, years and source origin	Objective/s	Participants	Concept/context	Method/cycles and stages/collection techniques/duration	Knowledge building	Social change
Hyde et al. (2009) Australia	To develop and implement a clinical decision‐making framework around the use of seclusion	*N* = n. a. Nurses of two acute inpatient wards (from nurse unit manager to enrolled nurse)	Decision‐making in the use of the seclusion Inpatient care	Action‐research/4 cycles of ‘plan, do, study, act’/Workshops, Discussion groups/Duration not specified	The only factor that was identified as influencing the decision to seclude a patient was safety. Two decision flowcharts were developed for the decision to confine and the decision to release	Improved seclusion records. The manager was involved in the review and follow‐up of seclusion cases
Borg et al. (2010) Norway	To examine the nature of practice models that are being developed in a Crisis Resolution/Home Treatment team incorporating the philosophy of open dialogue and the open lifeworld approach	*N* = 12 Crisis Resolution/Home Treatment team (one psychologist, two social workers and nine mental health nurses)	Evolution of clinicians using an open dialogue method Community care	Action research applying a cooperative inquiry perspective. /No cycles or stages are specified/Focus group/Duration not specified	A new model of clinical practice was conceptualized. The importance of being in the users' home environment and assessing the crisis situation there together with the individual and his or her family network by keeping the dialogue open, tolerating uncertainty and nurturing daily life issues	Unclear
Barret and Roberts (2010) United Kingdom	To improve the care of people with a dual diagnosis in one acute mental health care ward	*N* = 8 Five ward nurses, an academic with experience in dual diagnosis training, a service user trainer and a nurse consultant (the author)	Interventions by nurses to persons with dual diagnosis. Inpatient care	Practitioner‐based action research/Problem identification, action planning, implementing learning and reflection/Group sessions/Duration not specified	The need to improve the care of users with the dual diagnosis was identified. Specific learning needs and specific skills to engage more effectively were identified. The difficulty of staff shortages was detected. The effort to apply learning in practice was reflected on; different methods were proposed	Users changed their attitude about discussing substance use with nurses when previously they shied away from it. Nurses felt more able to rationalize their opposition to intoxicated patients and to successfully challenge the stance of some physician colleagues who had advised them to ban individuals from admission until sober
Chambers and Borschmann (2013) United Kingdom	To translate into practice a deeper, more evidence‐based understanding of the ‘lived experience of service users’ detained under the Mental Health Act	*N* = 33 19 detained service users, 14 staff members (five nurses, five health care assistants, two activity coordinators, one occupational therapist and one assistant neighbourhood manager)	Training program designed to optimize the hospitalization experience inpatient care	Action research/First action research cycle: phase 1 exploratory; Phase 2—intervention; Phase 3—evaluation/Semi‐structured interviews, focus group and surveys/27 months	Patient needs were identified. An educational intervention was designed for the staff. The participants found the training course motivating and valuable for their daily clinical practice. ‘a good refresher’	A greater sense of competence and confidence in interacting with service users was reported; also increased awareness of themselves and the needs of others, both service users and peers. Improved team relationships
Lackeman and Glasgow (2009) Ireland	To develop, implement and undertake an initial evaluation of a model of peer‐group clinical supervision for use in routine practice	*N* = 10 Psychiatric nurses	Peer‐group clinical supervision/Inpatient care	Collaborative action research/The first phase addressed establishing collaborative relationships with the service and participants and establishing the need and preferred form of clinical supervision. The second addressed the commissioning and provision of education and the initiation of supervision groups. The third phase involved the implementation and adaptation of supervision groups and the initial evaluation/Focus groups/5 months	Participants reported a greater number of roles that they had identified with or enacted since they began peer group supervision. These included teacher, counsellor, advisor, advocate, negotiator and protector.	Participants reported feeling more satisfied with their work. Most participants thought that peer experience in group supervision contributed to improvements in the care provided. All participants agreed that they felt more supported by their colleagues through group participation. Participants reported that they were more aware of taking on replacement roles
Onnela et al. (2014) Finland	To develop a professional practice model of mental health nurses in mental health promotion in a comprehensive school environment	*N* = n. a. Steering group (directors and coordinators in the health sector) to follow and evaluate the development process Mental health nurses to help evaluate the model and as the operators of the interventions School staff, parents and students	Mental health promotion Community care‐Scholar nursing	Participatory action research/Nine cycles of reflection, action and observation Workshops and reflective Journal Duration not specified	Interventions were developed at three levels: (a) universal level, targeting the entire school community. (b) selective level, focused on selected classrooms and groups. (c) indicated level, aimed at individual students with a low level of symptoms and their families	Group interventions improved bullying prevention, increased student knowledge and participation, improving the school and classroom environment. Developing interventions in collaboration with stakeholders increases community‐wide mental health promotion knowledge. Collaboration improves the implementation of interventions and supports the inculcation of interventions as part of the school function
Larkin et al. (2015) United Kingdom	To understand, and then improve, the experience of hospitalization during early psychosis	*N* = 21 (research phase) Six service users, six parents and nine inpatient staff. *N* = over 150 (Co‐design phase) Stakeholders: inpatient staff, community mental health staff, NHS managers, family members or service users	Improving the experience of hospitalization in people with early psychosis Inpatient care	Participatory action research, adapted Experience‐Based Co‐Design One cycle of eight stages in two phases: research phase and Co‐design phase/in‐depth interviews, feedback groups/ Duration not specified	Action plans were identified: 1. pathways in and out. 2. Provide staff with a rewarding and well‐supported role 3. Communication with families and service users 4. Recovery‐focused practice 5. Creating a positive environment for everyone 6. Recognize and share good practices across professions and services	Improvements that were relatively easy to implement were already underway in the first audit. Less progress was made on improvements that involved contributions from other departments or that involved strategic, budgetary or staffing commitments. The lack of continuity in high‐level support meant that responsibility for making improvements fell on a small group of individuals who were progressively more pressured and had little ‘empowerment’ when it was time to implement change
Chandley et al. (2014) United Kingdom	To explore the concept, and application, of ‘recovery’ in the care and clinical management of patients detained in one UK high‐security hospital	*N* = 40 20 patients 20 nurses	Improving recovery‐focused care inpatient care	Action research and ethnography/No cycles or stages are specified/Focus groups/3 years	The limitations and contradictions involved in employing recovery principles in forensic care were identified. Recovery resides in: safe contexts and in the relationships in the courtroom. A definition of recovery in the forensic context was identified. Factors influencing recovery in high‐secure care: Set A: protective factors; Set B: cultural factors; Set C: proactive approaches Set D: individual characteristics	There was an awareness of a trajectory from the past, to the present and into the future, although there was no single shared time axis. They spoke of trajectories from ‘being a criminal to going straight’, from ‘being a victim to becoming a survivor’, from ‘exclusion to inclusion’, and from ‘evil to redemption’, whilst very few conceptualized themselves as ‘sick’
Hutchinson and Lovell (2013) United Kingdom	To discuss the process of working with people who use statutory mental health services	*N* = 36 Six service users as researchers 30 service users as interviewees	The impact of mental illness on a person's identity Community care	Participatory action research/ No cycles or stages are specified/Training workshops, discussion group/peer support, semi‐structured interviews/3 years	Aspects of identity and its transformation process were identified: connection, recognized definitions of self and reciprocity	Exclude disease labels and work history. The group began to think and act differently, creating a shared language. Encourage teamwork as well as enhancing individual and group identities. Transform their identities, increase social experiences and regain control over one's own life
Moreno‐Poyato et al. (2019) Spain	To produce changes in the therapeutic relationship between clinical practice nurses and patients in psychiatric units	*N* = 9 Nurses, 4 men and 5 women	Improve the nurse–patient therapeutic relationship/ Inpatient care	Participatory action research/ 2 cycles with 4 stages each one Focus groups and reflective journals/6 months	Barriers to establishing a therapeutic relationship were identified, such as organization, lack of time in clinical practice, lack of motivation and job dissatisfaction. Strategies for change were identified: (a) A personalized nursing intervention space, (b) Knowledge updating (c) Reflection groups, which were subsequently implemented and evaluated	The nurses became aware of the theory in practice. The change in practice enhanced patient empowerment, involving the patient in the treatment objectives and tasks to be performed. The improvement and unification of the objectives for the whole team.
Croucher and Williamson (2013) United Kindom	To introduce a new system of risk assessment, based on traffic lights, into a community mental health team	*N* = 11 Team Leader, Community Psychiatric Nurses, Social Worker, Occupational Therapist, Housing Officer Support Worker	Mental health risk assessment and monitoring/ Community care	Action Research/ Look, Think and Act phases/ Focus groups/ Duration not specified	A traffic light system was designed to assess and monitor risk that the team could easily use	The traffic light was useful for better report writing, bringing clarity and guidance at planning meetings about risk levels and resource allocation. It served as a historical record too as well. The traffic light improved risk allocation if needed in collaboration with the care coordinator. This support to the care coordinator can reduce restrictions to care by introducing—when safe—positive risk elements, which can empower patients
Kidd et al. (2015) Australia	To explore the meaning of the term recovery to people with experience providing and receiving mental health services.	*N* = 11 Six consumers Four clinicians One career	Recovery process meaning /Community care	Cooperative enquiry/No cycles or stages are specified/Group Meetings/12 months	Systemic barriers in the recovery process were identified. The meaning of recovery as an ongoing quest in life was also identified, although it reflected variations for each participant.	Not identified
Salzmann‐Erikson (2017) Sweden	To describe the process of a team development project considering ward rule issues, and to develop a working model to empower staff in their daily in‐patient psychiatric nursing practices	*N* = 26 One unit manager, 11 male staff (two of whom were registered nurses and nine psychiatric aides) and 14 women (Eight of whom were registered nurses and six psychiatric aides)	Improve ward rules/ Inpatient care	Participatory Action Research/ Four phases: problem identification, planning, action and reflection / Group discussions, observations and field notes/9 months	More than 100 specific warding rules were identified. A framework was developed that embraced the (1) Safety Principle, (2) Structure Principle and (3) Interaction Principle. The principles were linked to normative guidelines and applied ethical theories: deontology, consequentialism and ethics of care	The ward rules were relaunched in favour of adopting the new principles. The working model reminded staff of the principles, empowered their professional decision making, decreased collegial conflicts due to greater acceptance of individual decisions, and generally improved well‐being at work. In addition, the work model also enabled staff to find support for their decisions based on the principles grounded in the ethics of totality
Vantil et al. (2020) Brazil	To describe risk management implementation in the safety of patients with mental disorders through action research	*N* = 13 Five nurses, two social workers, three psychologists, one speech therapist, one pharmacist and one physician	Improving patient safety/Inpatient care	Participatory Action Research / 12 phases: I. Exploratory phase; II. Research topic; III. Locating the problem; IV. Theory placement; V. Hypothesis; VI. Seminars; VII. Field observation, sampling and qualitative representativeness; VIII. Data collection; IX. Learning; X. Informal/formal knowledge; XI. Action plan; XII. External reporting/ Group meetings (8 in group A, 8 in group B, 1 consensus)/6 months	Priority identification and the risk factors that involved the safety of patients with mental disorders 7 protocols were developed: 1. Patient Correct Identification; 2. Hand Hygiene; 3. Violence Prevention; 4. Prescription and Safe Administration of Medications; 5. Patient Evasion Prevention; 6. Preventing Restraint Injury; and 7. Fall Prevention. the textual proposal of software for internal management of incident reports	Better organization of the practice, guaranteeing the professional that actions were standardized based on the principles of patient safety. Greater satisfaction for the nursing team and patient, as well as greater safety in the performance of procedures, reducing risks for professionals and patients

### 3.2 Use of action research in mental health nursing

The majority of the studies included in the review claimed to use either action research (*k* = 4) (Chambers et al., 2013; Chandley et al., 2014; Crouche & Williamson, 2013; Hyde et al., 2009) or participatory action research (*k* = 8) (Clements, 2012; Hutchinson & Lovell, 2013; Lange, 2011; Larkin et al., 2015; Moreno‐Poyato et al., 2019; Onnela et al., 2014; Salzmann‐Erikson, 2017; Vantil et al., 2020). Other studies also employed collaborative action‐research (*k* = 1) (Lakeman & Glasgow, 2009), practitioner‐based action research (*k* = 1) (Barrett & Roberts, 2010) and action research applying a cooperative inquiry perspective (*k* = 2) (Borg et al., 2010; Kidd et al., 2015). Most studies described the cycles and stages of the action research process that guided the process. Thus, the different approaches described in the studies were grouped into four stages: (1) Exploring and identifying the problem, (2) Planning action, (3) Implementing action and (4) Evaluating (Table 2). However, in six of the studies, no explicit reference was made to research cycles and stages (Borg et al., 2010; Chandley et al., 2014; Clements, 2012; Hutchinson & Lovell, 2013; Kidd et al., 2015; Lange, 2011).

**TABLE 2:** Stages of action research in mental health nursing studies

Stages of action research		Hyde et al. (2009) and Lackeman and Glasgow (2009)	Barret and Roberts (2010), Moreno‐Poyato et al. (2019) andSalzmann‐Erikson (2017)	Chambers et al. (2013) and Vantil et al. (2020)	Onnela et al. (2014)	Croucher and Williamson (2013)
Reflection process	Exploring and identifying the problem		Problem identification	Exploring	Reflecting	Look
	Planning action	Plan	Action planning			Think
	Implementing action	Do	Implementing/action	Intervention	Action	Act
	Evaluating	Study and act	Reflection	Evaluation/Divulgation	Observation	

Concerning the research methods, in all 16 studies group meetings were held in the form of focus groups, workshops and other denominations. However, in four of the studies, interviews were also conducted (Chambers et al., 2013; Clements, 2012; Hutchinson & Lovell, 2013; Larkin et al., 2015) and in two cases reflective journals were used (Moreno‐Poyato et al., 2019; Onnela et al., 2014). Other data collection methods and techniques used included photographs and text pieces (Clements, 2012) or observations and field notes (Salzmann‐Erikson, 2017).

### 3.3 Topics of interest for improving mental health nursing care through action research

**FIGURE 2:**
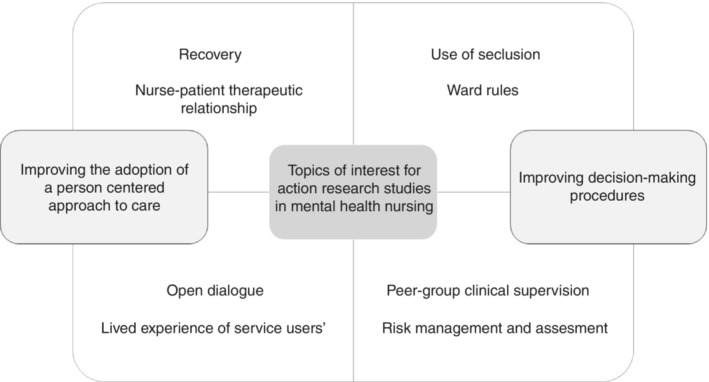
Topics of interest for improving mental health nursing care through action research.

Two categories were identified: improving the adoption of a person‐centred approach to care and improving decision‐making procedures (Figure 2). The category improving the adoption of a person‐centred approach to care included topics of studies that aimed to improve care from the people's experience. In this category, recovery‐focused care was the most addressed topic (Chandley et al., 2014; Clements, 2012; Kidd et al., 2015; Lange, 2011). For example, in the study by Kidd et al. (2015), systemic barriers in the recovery process were identified by people with experience in providing and receiving mental health services. Another topic of interest was to improve care by understanding the lived experience of service users (Chambers et al., 2013; Hutchinson & Lovell, 2013; Larkin et al., 2015). In this regard, the study by Hutchinson and Lovell (2013) is noteworthy, as users acted as interviewers of other users to identify the impact of the mental disorder on the person's identity. In this same category, we also identified papers aimed at improving other topics such as the nurse–patient therapeutic relationship (Moreno‐Poyato et al., 2019), the evaluation of the open dialogue model (Borg et al., 2010) and the promotion of mental health in the school setting (Onnela et al., 2014). The category improving decision‐making procedures included those topics that sought to improve more specific aspects of mental health care and that responded to the attempt to structure and operationalize procedures for decision‐making. In this sense, risk management and assessment of mental health were dealt with in two studies (Crouche & Williamson, 2013; Vantil et al., 2020). Here, the contribution made by Crouche and Williamson (2013) stands out, by introducing a new risk assessment system in a community mental health team. Other topics included in this category were the use of seclusion (Hyde et al., 2009), ward rules (Salzmann‐Erikson, 2017) and peer‐group clinical supervision (Lakeman & Glasgow, 2009). For both categories, all studies reviewed aimed to either improve nursing care (Barrett & Roberts, 2010; Chandley et al., 2014; Hyde et al., 2009; Moreno‐Poyato et al., 2019; Onnela et al., 2014) or improve the clinical practice of teams (Borg et al., 2010). About the improvement of nursing care, the study by Moreno‐Poyato et al. (2019), which implemented an evidence‐based intervention to improve the nurse–patient therapeutic relationship in mental health units, should be highlighted. Borg et al. (2010) were able to conceptualize a new model of clinical practice in a home treatment team centred on open dialogue.

### 3.4 Action research as a source of knowledge in mental health nursing

In all the studies included in the review, it was possible to clearly identify the knowledge generated. In this regard, the first aspect highlighted in all the articles was that the method made it possible to identify the meaning that the participants gave to the study phenomenon to be improved. Thus, in most cases, the participants' needs and priorities were identified (Barrett & Roberts, 2010; Chambers et al., 2013; Chandley et al., 2014; Hyde et al., 2009; Lakeman & Glasgow, 2009; Lange, 2011; Moreno‐Poyato et al., 2019; Vantil et al., 2020) together with the difficulties and barriers they encountered in practice (Barrett & Roberts, 2010; Chandley et al., 2014; Kidd et al., 2015; Moreno‐Poyato et al., 2019). Additionally, most of the studies described specific, adapted and efficient strategies to improve the topic of interest (Barrett & Roberts, 2010; Chambers et al., 2013; Chandley et al., 2014; Hyde et al., 2009; Larkin et al., 2015; Moreno‐Poyato et al., 2019; Onnela et al., 2014). Other studies also identified new practice models, such as the incorporation of the open dialogue in a home treatment team model (Borg et al., 2010) or the development of a professional practice model for mental health nurses working in mental health promotion in a school setting (Onnela et al., 2014).

### 3.5 Action research as a driver of change in mental health nursing

The first aspect that stands out in most of the studies as a product of change is the participants' awareness of the topic of interest (Barrett & Roberts, 2010; Borg et al., 2010; Chambers et al., 2013; Chandley et al., 2014; Clements, 2012; Hyde et al., 2009; Lakeman & Glasgow, 2009; Moreno‐Poyato et al., 2019). In addition, the participants improved their attitude and level of knowledge, acquiring greater capacity and competence in relation to the phenomenon they were trying to change (Barrett & Roberts, 2010; Chambers et al., 2013; Lakeman & Glasgow, 2009; Moreno‐Poyato et al., 2019; Onnela et al., 2014). Changes in teamwork were also identified. Participants felt more supported and relationships in the team were improved (Chambers et al., 2013; Lakeman & Glasgow, 2009; Moreno‐Poyato et al., 2019; Salzmann‐Erikson, 2017), involving managers (Crouche & Williamson, 2013; Hyde et al., 2009) and improvements in the consensus of objectives (Hutchinson & Lovell, 2013; Hyde et al., 2009; Moreno‐Poyato et al., 2019). In this sense, the process has been found to generate motivation and satisfaction (Chambers et al., 2013; Moreno‐Poyato et al., 2019; Vantil et al., 2020), providing security and empowering the participants in the project (Barrett & Roberts, 2010; Clements, 2012; Kidd et al., 2015; Moreno‐Poyato et al., 2019; Salzmann‐Erikson, 2017; Vantil et al., 2020).

## 4 DISCUSSION

The purpose of this review was to locate and describe the available evidence related to the use of the action research method in the context of mental health nursing. First, it should be noted that it is a method used to improve aspects related to mental health nursing in both inpatient and community care settings. These findings confirm that action research is useful in any context that aims to improve aspects of health (Cordeiro & Soares, 2018). In addition, it is important to point out that it has the capacity to converge stakeholders in the phenomenon to be improved. This fact is especially relevant in current international health research policies, where the active participation of citizens in all steps of the research process is promoted, both in the development of projects and in the involvement of citizens in the dissemination and consumption of science (Vohland et al., 2021). However, in the case of mental health nursing, only half of the studies included users. This is also the case in other contexts, where there are still a small number of studies in which equal groups are formed between researchers and other stakeholders, not only in the design of the study but also in the selection of participants and data collection and analysis (Effendy et al., 2022; Wiles et al., 2022).

From a methodological perspective, it is important to emphasize that although there are different approaches to action research (Rowell et al., 2017), the results of the review indicate that in the field of mental health nursing the most commonly used method is Participatory Action Research. This may be due to the need to work in a participatory fashion, with researchers and stakeholders side by side to address a shared concern which is the driver of the process of change (Kemmis & Mctaggart, 2008). Another outstanding aspect of the results from a methodological point of view was that most of the studies described the cyclical designs used and this made it possible to elaborate a classification of the common stages identified. However, over one‐third of the studies did not specifically describe the cyclical process of the research, whereas some did so in a vague manner, although one of the characteristics of action research is the cyclical nature of the design and process (Casey et al., 2022; Rowell et al., 2017). This finding may explain why, in many of the studies, the results related to the change produced were not clearly identified. In this sense, the lack of description of the stages of action research is an important limitation for assessing the quality of the study report (Casey et al., 2022; Waterman et al., 2001).

The results indicate two major foci for which the action research method has been used in the area of mental health nursing: improving person‐centred models of care and improving decision‐making procedures. As in other contexts of nursing practice, heterogeneous group participation allows issues of collective concern to be addressed (Cordeiro & Soares, 2018; Cusack et al., 2018), whether it deals with issues related to the care model (Blackwell et al., 2017; Rönnerhag et al., 2019), or if it focuses on improving more specific aspects related to day‐to‐day decision making (Cerulus et al., 2021; Jokiniemi et al., 2021).

As a source of knowledge, the results of the review point to the usefulness of action research for exploring the meaning of the phenomenon of interest, making it easier for participants to identify what they need to improve and how they can improve it more efficiently (Casey et al., 2022). Thus, action research is a driver for change and improvement in the practice of mental health nurses. As in other nursing contexts, the findings point to the importance of awareness as a starting point for change (Bekkema et al., 2021; Cusack et al., 2018). This awareness allows the participants to improve their attitude and knowledge about the phenomenon of concern (Tolosa‐Merlos et al., 2021). This, together with the support received from the group, improves team relations, increasing motivation and satisfaction in the work environment (Afshar et al., 2020; Chen et al., 2021). Throughout the process, participants gain confidence and security, key aspects for the empowerment of participants (Alomari et al., 2020; Bekkema et al., 2021; Cusack et al., 2018).

### 4.1 Limitations

This review has some limitations. First, including the English language filter as an inclusion criterion may have resulted in studies that could not be reviewed. Second, it is possible that the search terms may have limited the scope of the identified articles. However, the list of search terms was constructed in an elaborate, multistep process. Third, the articles included were of varying quality. In this regard, the team decided not to remove any articles to be able to more precisely analyse and report these limitations in the use of action research. Nonetheless, this may have had an impact on the reliability of the results obtained in relation to other issues analysed. Fourth, only articles that presented results from the entire action research process were included. This may have meant that projects that have not published articles with complete results could not be reviewed. It is important to consider that many action research projects are represented in several articles; a single article may aim to highlight only one phase of the project, rather than all phases. However, the claims of the review were to provide knowledge about the use of action research in mental health nursing as a whole. Given the transformative nature of the method used in the included studies, the results obtained may be transferable to similar contexts and populations.

## 5 CONCLUSION

This review has identified and synthesized the available evidence on the use of action research in the context of mental health nursing. The findings demonstrate that Participatory Action Research is the most widely used action research method and that it is useful for enhancing models of care and improving decision‐making procedures, both in the hospital and community settings. Action research can improve aspects of nursing practice and team dynamics. However, it should be noted that the quality assessment of action research articles is complex and requires further research.

In light of the findings of this review, three key points are identified to improve the effectiveness and quality of the use of action research on mental health nursing care:

- The perspective of different stakeholders should be incorporated throughout the research process, from project design to the reporting of results. Action research includes the participation of different stakeholders and, therefore, incorporates a collaborative approach both in the identification of the problem and in the actions to foster change. In this sense, it allows all stakeholders to become aware of the issue of interest, directly applying knowledge in practice and obtaining satisfaction and empowerment. However, still, in the context of mental health nursing, stakeholders are not representatively included in all projects and all their phases.
- It is necessary to provide a detailed description of the cycles and stages of the whole process, together with the objectives to be achieved for each of them. The use of action research, as a method of a cyclical and transformative nature, requires that special attention be paid not only to the end results of the method but also to the results produced in the process. It is important that researchers adequately describe both the stepwise design carried out and the results obtained during the process.
- Clear reporting is warranted on the knowledge generated and the change produced throughout the action research process. Although the purpose of action research is to promote change, in some studies, the impact is not clearly reflected in the results section.

## AUTHOR CONTRIBUTIONS

Made substantial contributions to conception and design, acquisition of data, or analysis and interpretation of data: ARMP, DTM, AVR, APT, KE, RNM, RSP, RVF. Involved in drafting the manuscript or revising it critically for important intellectual content: ARMP, MSM, MRL, TLLC, JRM, MPLL. Given final approval of the version to be published. Each author should have participated sufficiently in the work to take public responsibility for appropriate portions of the content: ARMP, DTM, AVR, APT, KE, RNM, RSP, RVF, MSM, MRL, TLLC, JRM, MPLL. Agreed to be accountable for all aspects of the work in ensuring that questions related to the accuracy or integrity of any part of the work are appropriately investigated and resolved: ARMP, DTM, AVR, APT, KE, RNM, RSP, RVF, MSM, MRL, TLLC, JRM, MPLL.

## FUNDING INFORMATION

None.

## CONFLICT OF INTEREST

None.

### PEER REVIEW

The peer review history for this article is available at https://publons.com/publon/10.1111/jan.15463.

## Supporting information


Data S1.

Data S2.

## Data Availability

The data that support the findings of this study are available from the corresponding author upon reasonable request.
