# Supplementary material for: A systematic review on the use of action research methods in mental health nursing care
Source: J Adv Nurs. 2022 Oct 27;79(1):372–84. doi: 10.1111/jan.15463 (PMC10092408; doi:10.1111/jan.15463)
Supplement: Supplementary file 1 — Data S1. [file JAN-79-372-s002.docx]

**Supplementary file 1: Databases and search strategy**

| **Database** | **Search strategy** |
| --- | --- |
| Scopus | TITLE-ABS-KEY-AUTH(mental health OR psychiatric AND "action research") AND ( LIMIT-TO (SUBJAREA,"NURS" ) AND ( LIMIT-TO ( PUBYEAR,2020) OR LIMIT-TO ( PUBYEAR,2019) ( LIMIT-TO ( PUBYEAR,2018) OR LIMIT-TO ( PUBYEAR,2017) OR LIMIT-TO ( PUBYEAR,2016) OR LIMIT-TO ( PUBYEAR,2015) OR LIMIT-TO ( PUBYEAR,2014) OR LIMIT-TO ( PUBYEAR,2013) OR LIMIT-TO ( PUBYEAR,2012) OR LIMIT-TO ( PUBYEAR,2011) OR LIMIT-TO ( PUBYEAR,2010) OR LIMIT-TO ( PUBYEAR,2009)) |
| Pubmed | (mental health OR psychiatric) AND nursing AND "action research" Filters: 10 years |
| Web of Science | TS=((mental health OR psychiatric) AND nursing AND action research)  Refined by: RESEARCH AREAS: ( NURSING )  Time period: 2009-2020.  Databases: WOS, CCC, DIIDW, KJD, MEDLINE, RSCI, SCIELO. |
| CINAHL | ( mental health nursing or psychiatric nursing ) AND action research filter 10 years |
