# Supplementary material for: A systematic review on the use of action research methods in mental health nursing care
Source: J Adv Nurs. 2022 Oct 27;79(1):372–84. doi: 10.1111/jan.15463 (PMC10092408; doi:10.1111/jan.15463)
Supplement: Supplementary file 2 — Data S2. [file JAN-79-372-s001.docx]

**Supplementary file 2: Quality assessment of included studies**

The Joanna Briggs Institute Critical Appraisal Checklist for Qualitative Research

| **Author/year** | **1. Is there congruity between the stated philosophical perspective and the research methodology?** | **2. Is there congruity between the research methodology and the research question or objectives?** | **3. Is there congruity between the research methodology and the methods used to collect data?** | **4. Is there congruity between the research methodology and the representation and analysis of data?** | **5. Is there congruity between the research methodology and the interpretation of results?** | **6. Is there a statement locating the researcher culturally or theoretically?** | **7. Is the influence of the researcher on the research, and vice- versa, addressed?** | **8. Are participants, and their voices, adequately represented?** | **9. Is the research ethical according to current criteria or, for recent studies, and is there evidence of ethical approval by an appropriate body?** | **10. Do the conclusions drawn in the research report flow from the analysis, or interpretation, of the data?** | **Overall appraisal** |
| --- | --- | --- | --- | --- | --- | --- | --- | --- | --- | --- | --- |
| Barret and Roberts (2010) | Yes | Yes | Yes | Yes | Yes | No | No | Yes | Yes | Yes | 8 |
| Borg et al. (2010) | Yes | Yes | Yes | Yes | Yes | Unclear | Yes | Yes | Yes | Yes | 9 |
| Chambers et al. (2013) | Yes | Yes | Yes | Yes | Yes | Unclear | No | Yes | No | Yes | 7 |
| Chandley et al. (2014) | Yes | Yes | Yes | Unclear | Yes | No | No | Yes | No | Yes | 6 |
| Clements (2012) | Yes | Yes | Yes | Yes | Yes | Unclear | No | Yes | Yes | Yes | 8 |
| Croucher and Williamson (2013) | Yes | Yes | Yes | Yes | Yes | Yes | Yes | Yes | Yes | Yes | 10 |
| Hutchinson and Lovell (2013) | Yes | Yes | Yes | Unclear | Yes | Yes | Unclear | Yes | Yes | Yes | 8 |
| Hyde et al. (2009) | Yes | Yes | Yes | Yes | Yes | No | No | No | No | Yes | 6 |
| Kidd et al. (2015) | Yes | Yes | Yes | Yes | Yes | Yes | Yes | Yes | Yes | Yes | 10 |
| Lakeman and Glasgow (2009) | Yes | Yes | Yes | Yes | Yes | Yes | No | Yes | Yes | Yes | 9 |
| Lange (2011) | Yes | Yes | Yes | Yes | Yes | Yes | Yes | Yes | Yes | Yes | 10 |
| Larkin et al. (2015) | Yes | Yes | Yes | Yes | Yes | No | No | Unclear | Yes | Yes | 7 |
| Moreno-Poyato et al (2019) | Yes | Yes | Yes | Yes | Yes | Yes | Yes | Yes | Yes | Yes | 10 |
| Onnela et al. (2014) | Yes | Yes | Yes | Yes | Yes | Yes | No | No | Yes | Yes | 8 |
| Salzmann‑Erikson (2017) | Yes | Yes | Yes | Yes | Yes | Yes | Yes | Yes | Yes | Yes | 10 |
| Vantil et al. (2020) | Yes | Yes | Yes | Yes | Yes | Unclear | No | Yes | yes | Yes | 8 |
